# From Plasminogen to Plasmin: Role of Plasminogen Receptors in Human Cancer

**DOI:** 10.3390/ijms151121229

**Published:** 2014-11-17

**Authors:** Miroslava Didiasova, Lukasz Wujak, Malgorzata Wygrecka, Dariusz Zakrzewicz

**Affiliations:** 1 Department of Biochemistry, Faculty of Medicine, University of Giessen Lung Center, Friedrichstrasse 24, 35392 Giessen, Germany; E-Mails: miroslava.didiasova@biochemie.med.uni-giessen.de (M.D.); lukasz.wujak@mpi-bn.mpg.de (L.W.); 2 The German Center for Lung Research, 35392 Giessen, Germany; E-Mail: malgorzata.wygrecka@innere.med.uni-giessen.de

**Keywords:** actin, annexin 2, cytokeratin-8, enolase-1, plasmin, plasminogen, plasminogen receptor

## Abstract

Cell surface-associated proteolysis mediated by plasmin (PLA) is an essential feature of wound healing, angiogenesis and cell invasion, processes that are dysregulated in cancer development, progression and systemic spread. The generation of PLA, initiated by the binding of its precursor plasminogen (PLG) to the cell surface, is regulated by an array of activators, inhibitors and receptors. In this review, we will highlight the importance of the best-characterized components of the PLG/PLA cascade in the pathogenesis of cancer focusing on the role of the cell surface-PLG receptors (PLG-R). PLG-R overexpression has been associated with poor prognosis of cancer patients and resistance to chemotherapy. We will also discuss recent findings on the molecular mechanisms regulating cell surface expression and distribution of PLG-R.

## 1. Introduction

The plasminogen/plasmin (PLG/PLA) system is involved in various physiological as well as pathological processes mainly due to its ability to regulate pericellular proteolytic activity and thus cell motility [1]. A growing body of evidence suggests that dysregulation of any of the PLG/PLA system components may result in tumor growth and metastasis formation [2]. Indeed, overexpression of PLG-R including actin (ACT), enolase-1 (ENO-1), cytokeratin 8 (CK8) and annexin 2 (ANX2) has been associated with poor prognosis and resistance to chemotherapy of cancer patients. Therefore, PLG-Rs have become good diagnostic and prognostic markers, for instance, in breast, lung and pancreatic carcinomas [3,4,5]. Interestingly, many PLG-Rs are intracellular proteins with established functions in processes such as glycolysis, DNA packaging or cytoskeleton organization [6]. Their transport from the cytoplasm to the cell surface is necessary to accomplish their PLG-R function; however, most of them lack a signal sequence and thus cannot be translocated to the cell surface through the classical endoplasmic reticulum-Golgi pathway. Although the mechanism underlying PLG-R exteriorization is not fully understood, their translocation to and association with the cell surface may be induced by several stimuli [2,6]. An accumulating body of evidence indicates that targeting PLG-Rs bound to the cell surface may represent a promising therapeutic approach for the treatment of cancer patients. Hence, future investigations should address the molecular mechanisms underlying trafficking of PLG-Rs to the outer leaflet of the plasma membrane. In this review we will focus on the multifunctional role of the PLG/PLA system in the pathogenesis of human cancer and, in particular, we will discuss the mechanisms involved in the regulation of PLG-R trafficking and highlight novel/potential anticancer therapies targeting these molecules.

## 2. The PLG Activation System 

**Figure 1:**
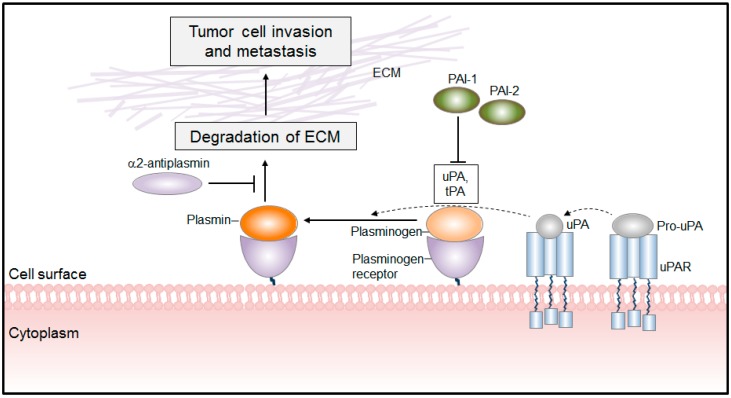
The mechanism of PLA-mediated tumor cell invasion and metastasis formation. Plasminogen binds to its receptors and is subsequently converted to plasmin by plasminogen activators (uPA, tPA). The cell surface-associated PLA facilitates tumor cell invasion by breaking down the ECM. The proteolytic activities of uPA and PLA are antagonized by PLG activator inhibitor-1 and -2 (PAI-1, PAI-2) and by α2-antiplasmin, respectively. ECM, extracellular matrix.

The zymogen PLG is secreted as a single chain glycoprotein by the liver, and circulates in the blood in an inactive form. It consists of an *N*-terminal activation peptide, five kringle domains and a serine-protease domain [7]. Kringle domains are responsible for substrate binding and interaction of PLG with cell surface proteins. Association of PLG with cellular receptors alters PLG conformation thereby facilitating its activation by the urokinase plasminogen activator (uPA) or tissue plasminogen activator (tPA) [7] (Figure 1). Notably, uPA is synthesized and secreted as a zymogen (pro-uPA) and gets activated upon binding to its cellular receptor, uPAR [8]. Thus, the efficient activation of PLG requires: (1) receptor bound uPA; (2) cell surface bound PLG; and (3) uPA-PLG interaction [7,9] (Figure 1). Of note, the binding of PLG and subsequent PLA generation are not restricted to the cell surface. Many components of the extracellular matrix, such as fibrin, lamin and fibronectin, are capable of binding PLG [9]. For instance, parallel adsorption of PLG and tPA onto fibrin results in efficient generation of PLA and thus promotes fibrinolysis, an important process in cancer progression [10].

Physiological regulation of the PLG/PLA system relies on numerous proteases and protease inhibitors. The major inhibitor of this cascade is α2-antiplasmin. Interestingly, this inhibitor blocks only the activity of free PLA, while leaving the cell surface-associated PLA intact [11]. The PLG/PLA system can also be regulated at the level of PLG activation by two serine protease inhibitors, plasminogen activator inhibitor (PAI)-1 and PAI-2 [11]. PAI-1 is an inhibitor of tPA and uPA, while PAI-2 exhibits inhibitory activity mainly toward uPA and is less effective against tPA [12].

The main function of PLA is clot dissolution and degradation of the extracellular matrix (ECM) proteins. However, PLA may also activate matrix-metalloproteinases (MMPs) such as MMP-1, MMP-3, and MMP-9 and growth factors including transforming growth factor beta (TGF-β), basic fibroblast growth factor (bFGF) and vascular endothelial growth factor (VEGF) [13]. Most of these molecules are trapped within the ECM in an inactive form [13]; proteolytic processing of ECM components liberates them and thus facilitates their binding to the cognate receptors [13]. Apart from its proteolytic activity, PLA may activate intracellular signaling pathways and thus affect cellular processes. It is well recognized that PLA induces neutrophil aggregation, hepatocyte proliferation, monocyte chemotaxis, migration of endotheliocytes and expression of proinflammatory [14] and growth-factor like genes [15] via activation of multiple signaling pathways including JAK1/STAT, ERK1/2 [14], p38 mitogen-activated protein kinase (MAPK) [16] and the nuclear factor-κB (NF-κB) [17]. Several receptors have been found to mediate PLA-induced cellular activities. These include ANX2 [18], protease-activated receptor (PAR)-1 and -4 [15,19], and integrins [7]. Binding of PLA to the cell surface-associated ANX2 leads to ANX2 cleavage initiating a proinflammatory signaling cascade in monocytes [18]. Activation of PAR-1 by PLA induces Cyr61 gene expression in fibroblasts [15] and cleavage of PAR-4 activates platelets [19]. Integrins were also demonstrated to serve as PLA receptors. It was reported that α_Ϻ_β_2_ integrin promoted survival of neutrophils in response to PLG stimulation [20], and, α_ν_β integrin stimulated PLA-triggered migration of endotheliocytes [7]. Thus, PLA together with its precursor PLG can control numerous cellular functions either through activation/degradation of the extracellular ligands or by stimulation/perpetuation of the intracellular signaling pathways.

### PLG/PLA System in Cancer

Degradation of the ECM is a crucial step in tumor cell invasion and thus metastasis formation. PLA is one of several proteases facilitating tumor cell motility by disrupting the basement membrane and stromal barriers [7]. Excessive PLA production is frequently observed in tumors and it is a direct consequence of augmented expression and activity of PLG activators [10]. Thus, PLA may affect a number of cell functions, which are frequently dysregulated upon tumorigenesis including cell adhesion, proliferation, migration and apoptosis [10]. In addition, PLA may regulate tumor development by its capability to regulate cancer nutrition, oxygen supply [21], formation of blood vessels [22], and activation of VEGF [13], a key mediator of angiogenesis [23].

The critical role of the PLG/PLA system in cancer biology is supported by *in vivo* studies utilizing PLG-deficient mice (*Plg^−/−^*). In the MMTV-PymT model of mammary gland carcinogenesis, PLG deficiency markedly reduced the number of spontaneous pulmonary metastases pointing towards the *in vivo* role of PLG in tumor spread [24]. Suppressed angiogenesis and decreased metastatic potential was also observed in *Plg*^−/−^ mice after intracarotid inoculations of melanoma cells [25]. Finally, implantation of uPA competent T241 fibrosarcoma cells into *Plg^+/+^* mice enhanced tumor growth and angiogenesis as compared to *Plg^−/−^* littermates further emphasizing the role of the PLG/PLA system in cancer biology [26].

The expression of uPA and its receptor uPAR was frequently found to be dysregulated in many types of human cancer and their high levels were reported to positively correlate with poor prognosis [2]. Recently, increased expression of uPA and uPAR was demonstrated in glioblastoma, breast, lung, gastric, ovarian, colorectal, liver and prostate cancer [2,27,28]. On the basis of these findings, numerous experimental approaches targeting PLG activators in the pro-proliferative diseases were initiated. Several studies demonstrated that inhibition of either uPA or uPAR diminishes the metastatic potential of transplantable tumor cell lines [29]. The mechanism by which uPA/uPAR promotes tumorigenesis is complex; nevertheless, activation of the intracellular signaling pathways initiated by the binding of uPA to uPAR seems to play a major role. uPA-uPAR interaction affects cell viability, proliferation and apoptosis of tumor cells [29]. These effects can be mediated by the epidermal growth factor (EGF) receptor since uPA/uPAR overexpressing cells are characterized by constitutive activation of EGF receptor. Activation of the EGF receptor leads to the imbalance between pro-apoptotic and pro-proliferative factors, in favor of the latter ones [30]. Besides its role in cell viability, uPA/uPAR interaction regulates invasive properties of cancer cells. This fact was confirmed by experiments demonstrating that downregulation of uPA and tPA inhibits invasion of glioma cells by decreasing phosphorylation of FAK, p38 MAPK, JNK and ERK1/2 as well as activity of phosphatidyinositol 3-kinase, AKT and the mTOR pathway [31]. Furthermore, a number of studies demonstrated the involvement of uPA/uPAR in the regulation of cell adhesion, for example, by the interaction with vitronectin [32]. Thus, binding of uPA to uPAR may promote tumor invasion and progression either by influencing the PLA-mediated pericellular proteolytic activity, which is important for cancer cells to invade surrounding tissue, or by activating intracellular signaling pathways leading to changes in cell adhesion and viability.

Similarly to uPA, tPA was also found to be overexpressed in glioblastoma, leukemia, liver, melanoma and pancreatic ductal carcinoma [29]. Stimulation of cancer cells with tPA was shown to induce their proliferation, most likely, by the mechanism involving ERK1/2, the EGF receptor and ANX2 [33]. However, other membrane proteins were found to be involved as well. For example, binding of tPA to low density lipoprotein receptor-related protein (LRP)-1, a scavenger receptor, known to regulate cell spreading, receptor-mediated endocytosis and lipid homeostasis [34,35] induced expression of MMP-9 in a MEK1 and ERK 1/2 dependent manner [36], contributing to ECM degradation, tumor progression and spreading [37].

In advanced cancers uPA activity is significantly increased and serves as a prognostic indicator of poor patient outcome [29,38]. This would suggest that the levels of plasminogen activator inhibitors, PAI-1 and PAI-2, are rather reduced in these pathological conditions. Surprisingly, higher rather than lower levels of PAI-1 were found in breast, gastric, glioma, lung, ovarian, cervical and renal cancer cells as compared to non-malignant cells [29,39,40]. To date, the molecular mechanism of this apparent paradox remains largely unexplained, raising concerns whether therapeutic strategies to suppress tumor growth and angiogenesis should be aimed at inhibiting or enhancing uPA-PLA mediated proteolysis. While some studies showed that PAI-1 is necessary for tumor growth, others indicated that PAI-1 has either no effect or is inhibitory [41]. Investigating the PAI-1 paradox in cancer, McMahon and colleagues demonstrated that the effect of PAI-1 on tumor growth and angiogenesis depends on its abundance [42]. The role of PAI-1 in cancer cell adhesion also remains controversial, with some studies demonstrating that overexpression of PAI-1 upregulates cell surface expression of integrins thereby enhancing tumor cell adhesive properties [43], and others showing that PAI-1-induced LRP-1 mediated endocytosis impairs tumor cell binding to ECM [44]. Studies focusing on the role of PAI-2 in cancer pathogenesis are less conflicting. In contrast to PAI-1, reduced levels of PAI-2 were found in breast, lung and renal cancer cells [45,46]. Complementary *in vivo* studies revealed that overexpression of PAI-2 in melanoma cells is associated with a strong inhibition of metastasis formation [47,48] supporting a suppressive role of PAI-2 in tumor growth and spread. These opposite effects of PAI-1 and PAI-2 may be explained by differences in their structures, which affect their binding to the ECM components and to LRPs [46].

## 3. Plasminogen Receptors (PLG-R)

### 3.1. Classification of PLG-R

PLG-Rs are a heterogeneous group of cell surface proteins, which bind both PLG and PLA [49]. PLG-Rs are expressed in prokaryotes and eukaryotes [6]. In vertebrates, PLG-Rs have been found in a variety of cells including monocytes/macrophages, fibroblasts, platelets, and endothelial, as well as carcinoma cells [6,50]. The majority of PLG-Rs belongs to the so-called “moonlighting proteins” which exhibit various functions at distinct cellular and extracellular compartments [6]. Simultaneous expression of different PLG-Rs accounts for total PLG binding capacity of the cell [10] and reaches approximately 10^5^–10^7^ binding sites per cell [51]. PLG binding to the cell surface is tightly controlled by trypsin-like proteases including plasmin and trypsin [52]. Numerous *in vitro* studies demonstrated that these enzymes may cleave cell-surface associated proteins thereby exposing their carboxyl terminal lysine residues for PLG binding. This augments the PLG binding capacity of the cell, finally leading to the increase in its pericellular proteolytic activity [53]. Carboxypeptidases (Cp), such as CpN or CpB, that eliminate plasminogen binding sites, may thus dampen cell surface-associated proteolysis [53]. Hence, lysine residues either at the *C*-termini or internal ones that require proteolytic processing prior to PLG binding, became a major, but not exclusive, characteristic of PLG-Rs. On the basis of these observations PLG-Rs have been divided into four classes. First class includes proteins possessing carboxy-terminal lysine residues. Here, (i) ENO-1 on monocytes, neurons, carcinoma cells, lymphoid cells, myoblasts [6,54] and pathogenic bacteria [55]; (ii) CK8 on carcinoma cells [56]; (iii) p11 on endothelial cells [57]; and (iv) glyceraldehyde-3-phosphate dehydrogenase (GAPDH) on bacteria are the best characterized ones [55]. The second class combines receptors that require proteolytic processing of a lysine residue prior to PLG binding. This group comprises ANX2 on endothelial cells and ACT on endothelial and carcinoma cells [6]. Next, the third class consists of PLG-Rs that lack *C*-terminal lysine residues, yet are able to bind PLG and facilitate its activation. This class includes the αIIbβ3 integrin on platelets, α_M_β_2_ integrin on phorbol myristate acetate (PMA)-stimulated neutrophils, amphoterin on cancer cells and GP330 on epithelial cells [11,20,58]. Finally, the fourth group of PLG-Rs is composed of molecules that bind PLG, but do not promote its activation. The best-characterized examples here are tissue factor (TF) and non-protein molecules composed of glycosphingolipid and sialic acid linked to sugar chains of gangliosides [52].

### 3.2. PLG-Rs in Cancer

Components of the PLG/PLA cascade, in particular PLG-Rs, play a pivotal role in acquisition of the metastatic phenotype [2]. Their involvement in cancer development and progression was demonstrated in various *in vitro* and *in vivo* studies [2,59,60]. The importance of PLG-Rs for metastasis formation was highlighted for the first time in an *in vitro* model that was developed to study paclitaxel-resistant variants of invasive human cancer cell lines [61]. Direct comparison of the cell surface proteins in super-invasive and non-invasive cancer cells revealed marked upregulation of numerous PLG-Rs including ENO-1, ANX2 and ACT in super-invasive cells, providing strong evidence for their involvement in cancer cell invasion [61]. Moreover, highly metastatic MDA-MB-231 breast cancer cells were found to possess increased capacity to bind and activate PLG as compared to non-metastatic cell lines MCF-7 and T-47D [51]. Applying techniques that preserve cell integrity and exclude the contribution of PLG-Rs that adsorb to the cell surface upon cell death, Stillfried *et al.* [62] demonstrated that the modest increase in the PLG-binding capacity of the cell may result in the dramatic increase of its pericellular proteolytic activity. Summarizing, all these observations provide the basis to consider PLG-Rs as diagnostic markers and potential new drug targets. ENO-1, ANX2, CK8 and ACT are the best-described PLG-Rs until now, which are linked to the pathogenesis of human cancer.

#### 3.2.1. Expression and Function of ENO-1 in Cancer

Enolase is a key glycolytic enzyme that catalyzes conversion of 2-phosphoglycerate into phosphoenolpyruvate in the cytoplasm [50]. Besides its role in glycolysis, ENO is transported from the cytoplasm to the cell surface where it acts as a PLG-R [63]. In vertebrates, this enzyme possesses three distinct subunits and can form homo- or heterodimers [50,52]. Whereas the αα isoenzyme of ENO, also referred to as ENO-1, is ubiquitously expressed, the ββ isoenzyme is found predominantly in muscles and the γγ isoenzyme is characteristic for nervous tissue [50,52]. ENO-1 was found to be overexpressed in more than 20 types of human cancer [64]. This finding can be partially explained by the Warburg effect [65], which describes the increase in anaerobic glycolysis under hypoxic conditions, a common feature of most solid tumors [65]. Apart from its function in glycolysis, a spliced variant of ENO-1, the myc-binding protein-1, was found to act as a transcription factor responsible for the regulation of *c-myc* protooncogene expression [66,67]. Despite these facts, an increasing body of evidence suggests that ENO-1, present on the outer leaflet of the plasma membrane of cancer cells [68], may also modulate pericellular proteolytic activity and thus contribute to cancer progression and metastasis.

Lung cancer is the leading cause of cancer-related death worldwide [69]. The prognosis of lung cancer is poor due to the fact that this disease can be symptomless in the early stage; therefore, most lung carcinomas are diagnosed at an advanced stage when distant metastases are already present. Based on histologic appearance and presumed cellular origin, lung cancer can be divided into two main classes: small cell lung cancer (SCLC) and non-small cell lung cancer (NSCLC) [70]. Small cell lung cancer is of neuroendocrine origin, while NSCLC is predominantly epithelial. Non-small cell lung cancer, which accounts for approximately 75% of all lung cancers, is divided further into adenocarcinoma, squamous cell carcinoma, and large cell carcinoma [70]. ENO-1 was found to be significantly overexpressed in effusion-derived tumor cells and tumor specimens of lung cancer [71,72]. Chang and colleagues extended these observations and showed that ENO-1 cell surface expression was higher in late and end stage NSCLC and negatively correlated with patient survival and disease recurrence [72]. Moreover, antibodies directed against ENO-1 were found in sera from NSCLC patients and were more prevalent in advanced stages of the disease [73].

Increased expression of ENO-1 was also found in breast cancer tissue [64,74]. Elevated titers of anti-ENO-1 antibodies in sera from breast cancer patients were described as well [75,76,77]. Similarly to lung cancer, breast cancer patients whose tumors were characterized by the elevated expression of ENO-1 had poor prognosis with greater tumor size, poor nodal status, and a shorter disease-free interval [60]. These findings are supported by the studies demonstrating increased ENO-1 cell surface expression in the super-invasive breast cancer cell line MB-435S-F/Taxol-10p4p generated from MDA-MB-435S-F cells [78], and by the reports showing elevated ENO-1 levels in the less metastatic breast cancer cell line MCF-7 in response to the chemotherapeutic agent 4-hydroxy-tamoxifen (4-OHT) [74]. Notably, expression of ENO-1 in MCF-7 cells positively correlated with the increased proliferation and resistance to 4-OHT. In agreement with these results, Tu and colleagues reported that knockdown of ENO-1 in human breast cancer cells results in suppression of cell proliferation and increased sensitivity to 4-OHT [74]. This suggests that targeting ENO-1 in breast cancer cells may be a novel therapeutic approach to overcome 4-OHT resistance.

Up-regulated ENO-1 levels were also observed in head and neck cancers [79]. Here, increased levels of ENO-1 correlated with poor prognosis and the development of disease recurrence. *In vitro* studies revealed that ectopic overexpression of ENO-1 in oral cancer cells promotes their proliferation, migration and invasion. Elevated expression of the ENO-1 downstream target, a proinflammatory cytokine CCL-20, was responsible for these effects [79].

Pancreatic ductal carcinoma (PDAC) is another tumor, characterized by increased cell surface expression of ENO-1 [4,80]. In contrast to other human cancers where ENO-1 acts as pro-oncogenic agent, in PDAC ENO-1 favors anti-tumor responses [4]. Dendritic cells (DC) are antigen-presenting cells, which are able to display foreign antigens on their cell surface and present them to T-cells [76]. Interestingly, Cappello and colleagues demonstrated that autologous DC, which were pulsed with recombinant ENO-1, increased T-cell proliferation and enhanced the production of interferon-γ (IFN-γ) [4], a well-described anti-tumor agent [81]. This resulted in antitumor activity, which was manifested by proteolysis of PDAC cells [4]. These observations imply that T cell-mediated anti-tumor response against ENO-1 may likely be induced *in vivo* and suggest that ENO-1 may be a promising candidate for immunotherapeutic approaches in PDAC. 

The vast majority of the studies dedicated to the role of ENO-1 in cancer biology describe changes in total ENO-1 expression and the impact of these alterations on cell proliferation, viability, and activation of gene transcription [59,82]. Limited number of reports differentiates between cytoplasmic and cell surface-associated ENO-1 and addresses the role of membrane-bound ENO-1 in tumorigenesis. Although some studies demonstrated increased ENO-1 cell surface expression in late and end stages of cancer, its contribution to tumor progression and metastasis formation still remains speculative and requires further investigations. Nevertheless, a recently published study shows the presence of ENO-1 at the sites of pericellular ECM degradation and its co-localization with uPAR and PLG on lung cancer cells [68]. The same group further demonstrates that blockage of cell surface-bound ENO-1 reduces PLA-dependent ECM breakdown and thus cell invasion resulting in diminished metastatic potential of lung cancer cells *in vivo* [68]. This observation strongly implies that targeting cell surface-associated ENO-1 may offer a novel therapeutic option for patients suffering from cancer.

#### 3.2.2. The Role of CK8 in Cancer

Cytokeratin 8 belongs to a group of 21 cytokeratins that form intermediate filaments in epithelial and cancer cells [83]. Similarly to ENO-1, CK8 is localized in the cytoplasm and on the cell surface. Upon externalization CK8 acts as a PLG-R [83]. The mechanism underlying transport of CK8 to the cell surface remains elusive. Release of intracellular proteins occurring upon cell damage accompanying tumorigenesis is believed to contribute to the extracellular pool of CK8. Interestingly, cell surface expression of CK8 was demonstrated only in cancer cells and not in epithelial cells from healthy individuals [83,84].

Elevated CK8 levels have been detected in sera from lung cancer patients [3]. Fukunaga *et al.* [3] demonstrated augmented expression of CK8 in sera from NSCLC patients as compared to the subjects suffering from SCLC. In addition, sera levels of CK8 were higher in the patients with advanced NSCLC than in the patients with early stage of the disease. Elevated levels of CK8 in sera from NSCLC patients were associated with tumor progression and decreased survival. These findings correspond well with the *in vitro* observations demonstrating marked impact of cell surface associated CK8 on invasion and metastatic potential of NSCLC cell lines [85]. Together, this implies that CK8 is preferentially expressed in NSCLC and may serve as a prognostic marker for NSCLC patients.

Numerous studies demonstrated positive correlation between CK8 expression and invasiveness of breast cancer cells [86]. Moreover, sera collected from breast cancer patients contained high levels of CK8 alone or in complex with another member of cytokeratin family, CK18. Histologically, CK8 expression patterns vary amongst different types of breast cancer and therefore are used to distinguish between lobular and ductal carcinomas [87]. In ductal breast carcinoma, CK8 staining displays a diffuse cytoplasmic pattern condensed at the border of the cell, whereas in lobular carcinoma it is localized to the perinuclear region [87]. Furthermore, heterogeneous expression patterns of CK8 have been observed within the same cancer, namely ductal breast carcinoma [88]. Here, three different patterns of CK8 staining have been distinguished: (i) diffuse cytoplasmic staining (ii) membrane and peripheral cytoplasmic staining and (iii) strong membrane and granular cytoplasmic staining. Interestingly, the highest number of CK8 positive cells has been found at the invasion front [89]. Collectively, all these findings imply that CK8 expression may be used for molecular classification of breast cancer.

Besides breast and lung cancer, abnormal CK8 expression has been linked to the pathogenesis of other human carcinomas including oral squamous cell carcinoma [89], erythroleukemia [90], pancreatic and colon cancer [91]. For example, it has been observed that the increased level of CK8 in sera from the patients suffering from esophageal carcinoma correlates with tumor volume and patient survival [92]. An interesting study by Singh *et al.* [93] suggested that elevated levels of CK8 in patient sera may induce transformation of normal epithelium into esophageal cancer. This study examined the subjects who have a higher risk to get esophageal carcinoma due to poor nutritional status, consumption of tobacco products or alcohol intake and the subjects who are at low risk of becoming sick. Comparison of non-cancerous esophageal tissue between these two groups revealed significantly higher CK8 levels in sera of the subjects belonging to the high risk group. In addition, when esophageal tumors from the high and the low risk group were compared, less advanced tumors displayed significantly elevated CK8 levels in serum. These observations strongly suggest that the presence of CK8 in sera of patients is a cause rather than a consequence of esophageal cancer [93].

The important role of CK8 in the malignancies of skin and epidermis has been demonstrated as well [94]. Mice overexpressing CK8 in epidermis developed severe epidermal and hair follicle dysplasia leading to the development of areas of neoplastic transformation. These findings are in line with *in vitro* data showing that CK8 is capable of driving the transformation of skin tumors from a benign to malignant invasive phenotype [94].

#### 3.2.3. The Expression and Function of ANX2 in Cancer

Annexin 2 is phospholipid-binding protein, which is involved in diverse cellular activities, such as cell motility, cytoskeleton rearrangement, endo-/exocytosis and fibrinolysis [95]. This protein acts as PLG-R at the cell surface of endothelial and tumor cells [96,97]. ANX2 is composed of three regions (i) *N*-terminal, which binds p11 protein (ii) central, which interacts with phospholipids and (iii) *C*-terminal, which binds PLG [98,99]. Interestingly, recent data suggest that ANX2 does not bind PLG directly, but it rather participates in the transport of S100A10, a PLG regulatory protein, to the cell surface [53,100]. The studies addressing the role of ANX2 in cancer pathogenesis are controversial; however, there is a growing body of evidence suggesting that ANX2 may regulate cancer cell behavior and thus tumorigenesis. 

It is well-established that ANX2 overexpression predicts poor prognosis and survival of the patients with lung cancer [5]. The positive correlation between ANX2 levels and prognosis of cancer was described for NSCLC [5], lung squamous carcinoma and lung adenocarcinoma [101]. Furthermore, a direct involvement of ANX2 in the growth of lung cancer has been recently demonstrated [15]. Proteomic analysis of primary cancer tissue from NSCLC patients and matched lymph node metastatic tissue confirmed elevated levels of ANX2 in metastatic lymph nodes. In addition, ANX2 levels in patients with advanced clinical stage of NSCLC correlated with poor overall survival [15]. Accordingly, direct impact of ANX2 on cell proliferation *in vitro* and tumor growth *in vivo* was demonstrated [5]. To decipher the mechanism of ANX2 action, Wang *et al.* [5] proposed a novel model in which ANX2 works as a signal transducer facilitating cell cycle arrest through the regulation of p53 in NSCLC cells.

In breast carcinoma, an increase of ANX2 cell surface expression was reported in several studies and it was strongly correlated with an invasive phenotype of the tumor. It seems that ANX2 regulates breast cancer cell behavior by the mechanism involving PLG/PLA activation [102]. Preincubation of breast cancer cells with an anti-ANX2 antibody or angiostatin, a well-characterized ANX2 inhibitor, significantly impaired PLA generation and consequently reduced cancer cell invasion and migration [103]. These findings are supported by the studies demonstrating that increased cell migration of MDA-MB-231 cells depend on ANX2-mediated tPA binding and PLG activation [104]. Furthermore, ANX2, via its ability to control PLA formation, may regulate angiogenesis and thus contribute to the pathogenesis of breast cancer as well [103,104]. Notably, PLA can activate VEGF-C and VEGF-D, growth factors critically involved in the regulation of angiogenesis and lymphangiogenesis [105].

Numerous studies investigated correlations between the subcellular localization of ANX2 and aggressiveness and a clinical stage of prostate tumor [18,106]. Although many of these investigations suggest an essential function of cell surface-expressed ANX2 in tumor progression, the role of ANX2 in the pathogenesis of prostate cancer still remains controversial. Detailed analysis of ANX2 expression in prostate carcinomas revealed reduced ANX2 levels in cancer cells and even lack of its expression in advanced stages of the disease [18,106]. Reduced level of ANX2 was also observed in seven different prostate cancer cell lines. In those cells, ANX2 was localized to the cytoplasm and to the submembrane regions. Decreased levels of ANX2 correlated with an aggressive phenotype of cancer cells. Accordingly, re-expression of ANX2 inhibited migration of cancer cells without affecting their proliferation and apoptosis [106].

Studies focusing on the expression of ANX2 in pancreatic cancer are more consistent. Elevated levels of ANX2 were found in pancreatic adenocarcinoma cell lines, and in primary and metastatic tumors [107]. Furthermore, increased cell surface expression of ANX2 and its interacting partner S100A6 was noticed in patients suffering from pancreatic cancer [108]. Levels of cell surface-bound ANX2 positively correlated with advanced stages of the cancer and were dependent on high cytoplasmic expression of S100A6. In accordance with these findings, depletion of S100A6 resulted in reduction of ANX2 expression and consequently decreased motility of pancreatic cancer cells. Recent studies revealed the importance of ANX2 posttranslational modifications for its transport to the cell surface and thus invasive and metastatic potential of cancer cells, supporting previously published observations [109].

An association between abnormal ANX2 expression and progression of cancer was also observed in patients with renal [110,111] and ovarian carcinoma [112]. Both of these cancers displayed strong cell surface ANX2 staining. In addition, ANX2 was detected in supernatants of ovarian cancer and peritoneal cell co-cultures suggesting active release of this molecule from the intracellular compartment to the extracellular milieu [112].

To address the contribution of ANX2 to tumorigenesis Sharma *et al.* [113] demonstrated that administration of a neutralizing antibody directed against cell-surface associated ANX2 inhibits growth of human breast tumor in a xenograft model and that this effect depends, in part, on the attenuation of the neoangiogenic potential of tumor. This study strongly suggests that interference with ANX-2-mediated pericellular proteolytic activity may provide a novel strategy for specific inhibition of neoangiogenesis in human breast cancer.

#### 3.2.4. Expression and Function of ACT in Cancer

The ACT cytoskeleton is a dynamic complex of numerous proteins responsible for maintaining cell shape and cellular movement. Based on the molecular structure and tissue distribution, ACT isoforms have been classified into: (i) β and γ cytoplasmic (ii) α skeletal and α cardiac and (iii) α and γ smooth muscle isoactins [114]. Noteworthy, only cell surface expressed β and γ-ACT have been described to participate in PLA formation [115,116], and thus in tumorigensis. Overexpression of ACT was reported in different cancer cell lines and various types of human cancer [117,118,119]. Moreover, ACT levels were found to correlate with a more invasive phenotype [120,121].

Medullary carcinoma of the breast (MCB) is a rare morphologically distinct subtype of breast carcinomas, which has a more favorable prognosis than other types of breast cancer. One of the hypotheses explaining the biological basis for a favorable prognosis of MCB patients implies the involvement of lymphocytes infiltrating tumor stroma and restraining tumor growth [122]. Interestingly, immune responses of B and T cells infiltrating tumor stoma were found to be directed against β-ACT [123]. Anti-β-ACT immune responses are due to the exposure of β-ACT on the cell surface of the apoptotic MCB cells [124]. This renders β-ACT immunogenic and induces auto-antibody production.

Cytoskeletal ACT was also demonstrated on the cell surface and in cell culture supernatants of human lymphoid cells and lymphocytes undergoing blastogenic transformation [125]. Interestingly, leukemia cells were found to release actin into culture medium without loss of their viability. In addition, ACT released from cancer cells was not reabsorbing back to the membrane of these cells [126]. At present, it is questionable whether ACT released into supernatants may promote tumorigenesis. However, it is well-established that PLA formation is not restricted to the cell surfaces and ACT released from the cancer cells may potentially participate in local proteolysis, thus supporting tumor spread.

Actin has also been identified on the cell surface of prostate cancer cells [116], however, its role in tumor progression is not fully deciphered. On one side, ACT was shown to bind PLG and to participate in PLA formation, thus favoring angiogenesis. On the other side, a product of PLA autoproteolysis, angiostatin, was found to inhibit cell migration, proliferation and angiogenesis [127]. These studies highlight a dual role of cell surface-associated ACT in the pathogenesis of prostate cancer.

Actin polymerization and its reorganization into extensions such as filopodia and lamellipodia is a crucial process regulating cancer cell invasion [128]. Lamellipodia are the cell surface structures formed by the concentrated filaments of actin in the leading edge of the cell and are important for cell migration. Filopodia are also assembled from the cell surface actin filaments, but they are present over the whole cell surface serving as sensors of the environment [129,130]. Treatment of breast cancer cells with resveratrol, a grape polyphenol, which is thought to be a cancer preventive, inhibited cancer cell invasion by changing ACT filament organization from lamellipodia into filopodia [130]. Thus, ACT may contribute to tumor progression either via its ability to facilitate PLA formation or via its capability to modulate cytoskeleton organization and thus cell motility. Whether ACT present in lamellipodia may concentrate proteolytic activity through the regulation of PLA formation needs further investigation.

Abnormal organization of ACT into microfilaments seems to be one of the main features of colon carcinoma as well. High levels of ACT were reported in colon adenocarcinoma, and were correlated with increased motility of these cells [131]. Interestingly, the subcellular localization of ACT was dependent on the cell phenotype. Within colon carcinoma, two cell phenotypes were distinguished: (i) rounded with ACT present in the ring-like-form under the membrane; and (ii) elongated, with ACT concentrated in the leading edge. The biological relevance of this phenomenon is unclear; however both of these cell phenotypes were associated with increased invasion of cancer cells: elongated cells, by concentration of proteolytic activity on the leading edge; and rounded ones, by propagation of cell movement by a mechanism that does not require pericellular proteolysis [121]. Collectively, all these findings imply that dysregulated expression of ACT and its abnormal compartmentalization determine invasiveness of cancer cells [117].

**Figure 2:**
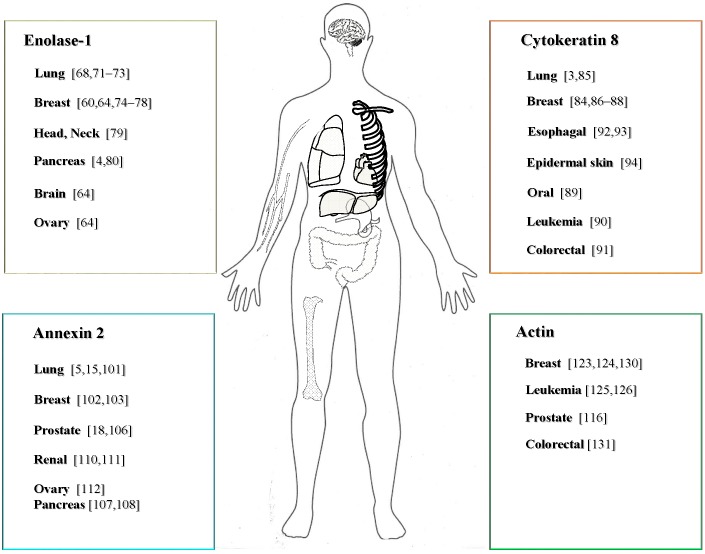
Contribution of selected PLG-R to the development and progression of cancer.

Altogether, dysregulated expression of PLG-Rs seems to be a reliable indicator of the disease stage and patients survival in several types of tumor (Figure 2). Furthermore, reports addressing the contribution of cell-surface-associated PLG-Rs to cancer biology allow us to speculate that PLG-R-dependent pericellular proteolytic activity may markedly influence metastatic potential of cancer cells. However, further studies focusing on the compartment specific expression of PLG-Rs in different types/stages of cancer as wells as on the mechanism responsible for their exteriorization are needed.

## 4. Mechanism of PLG-R Trafficking

“Moonlighting proteins” may perform more than one function depending on their subcellular localization. They are primarily expressed in the cytoplasm or nucleus, and regulate processes such as metabolism, DNA packaging or cytoskeleton organization [59,98,132,133]. Under stimulatory or pathological conditions PLG-Rs translocate from the cytosolic pool to the outer leaflet of the plasma membrane where they bind PLG, thereby contributing to its activation. The majority of PLG-Rs lacks a signal sequence that could direct them across the classical endoplasmic reticulum-Golgi secretory pathway, therefore, a “non-classical” membrane trafficking was proposed [134,135]. Although the mechanism of the transport of the PLG-Rs is not fully understood, studies reporting stimuli-dependent cell surface expression of PLG-Rs in various cell types have recently been published. For instance, it was demonstrated that one of the most potent inflammatory stimuli, lipopolysaccharide (LPS), may induce translocation of ENO-1 to the cell surface of monocytes, thereby increasing their pericellular proteolytic activity and invasive potential [136]. A similar observation was reported upon treatment of monocytes with IFN-γ in combination with vitamin D3 [133]. Additionally, the temperature was found to stimulate transport of ANX2 to the cell surface of human umbilical vein endothelial cells (HUVEC) contributing to the increased cell surface-associated fibrinolytic activity [137].

It has been suggested that a degradative phase event in cellular apoptosis can elevate the expression of PLG-Rs including ENO-1 on the cell surface of monocytes [138,139]. In line with these findings, enhanced PLG binding was observed on apoptotic and necrotic cells [139]. PLG-Rs are more abundant in intracellular compartments as compared to the cell surface, thus, a few dying cells can change overall PLG-binding capacity of the whole cell population. Direct comparison between viable and non-viable cells confirmed that dead cells bind PLG 100-times more than intact ones [51]. Together, all these findings strongly indicate that there is a large cytosolic pool of “silent” PLG-binding proteins, which can be rapidly exteriorized under inflammatory, apoptotic or other pathological conditions. The mechanism which docks PLG-R on the cell surface remains unclear, however, it seems that anionic phospholipids of the plasma membrane, such as phosphatidylserine (PS), may play an important role in this process [140,141]. PS is normally restricted to the inner leaflet of the plasma membrane, but it may be exposed on the cell surface upon monocyte/macrophage differentiation or cell apoptosis thereby increasing PLG binding capacity of the cell [141].

Although many stimuli potentiate the cell surface expression of PLG-Rs, the intracellular factors, which are essential for PLG-R translocation, as well as the molecular mechanism responsible for the transport of the cytosolic molecules through or across the cell membrane remain unknown. It seems that posttranslational modifications play an important role in the trafficking process as well as in the regulation of PLG-binding capacity of cell-surface-associated receptors. Several posttranslational modifications of PLG-R including phosphorylation, acetylation, methylation and nitration have previously been described and associated with malignant diseases [50,142,143]. For instance, phosphorylation of ANX2 was found to be required for its cell-surface expression in response to temperature stress [109]. Inhibition of tyrosine protein kinases or mutation of tyrosine 23 to alanine blocked ANX2 externalization, consequently decreasing PLA generation and pancreatic ductal adenocarcinoma invasion [109,137]. As heat stress-induced ANX2 translocation requires the presence of p11 (S100A10) [137], it is tempting to speculate that assembly of protein complexes is an essential step in transporting PLG-Rs across the cell membrane independently on the classical endoplasmic reticulum-Golgi secretory pathway. This notion is well in line with a recently published study demonstrating that caveolae-associated ANX2 along with caveolin-1 (Cav-1) are needed for the transport of cytosolic ENO-1 to the surface of breast cancer and monocytic cells [82]. Thus, it can be proposed that ENO-1 translocation depends on its association with intracellular caveolae-like vesicles or plasma membrane caveolae [82]. Another mechanism that may regulate cell surface expression of the PLG-R relies on extra- and intra-cellular calcium content. An increase of the cell surface expression of histone H2B, another PLG-R upon monocyte/macrophage differentiation was shown to be Ca^2+^ dependent and required active L-Type Ca^2+^ channels [141]. ANX2 externalization was also mediated by extra- and intra-cellular Ca^2+^ levels. Valapala *et al.* [144] suggested that Ca^2+^-triggered transport of ANX2 to the cell surface is a multistep process composed of a sequence of events in which elevated levels of Ca^2+^ mobilize cytosolic ANX2 to the cholesterol-enriched domains of the plasma membrane called lipid rafts, and facilitate its secretion to the extracellular milieu in the form of exosomes. As ANX2 interacts with ENO-1 [82], and ENO-1 is also released in the form of exosomes [19,145], it is tempting to speculate that these two proteins may use a similar way to be transported across the cell membrane. Thus, the exosomal pathway could be one of the mechanisms responsible for the transport of PLG-R to the extracellular milieu. This is of particular interest as exosomes were found to facilitate tumor growth, metastasis formation, and the development of drug resistance by carrying a large array of proteins and nucleic acids possessing oncogenic properties [146]. Hence, exosome-associated-PLG-R could be taken up by recipient non-malignant cells and, consequently, alter pericellular proteolytic activity, intracellular signaling pathways and activation status. Yet, the role of PLG-R in cell-to-cell communication process upon tumorigenesis awaits further investigation.

## 5. Conclusions and Final Remarks

In conclusion, various components of the PLG/PLA system such as uPAR, uPA or PAI-1 have already been implicated in different stages of tumorigenesis. However, a growing body of evidence suggests that dysregulated expression and cellular localization of PLG-R may be associated with the development and progression of different types of human cancer as well (Figure 2). This notion is supported by the recently published studies demonstrating that PLG-R may serve as prognostic markers in numerous malignant diseases, and that antibodies directed against PLG-R can be used as adjuvant in anticancer therapies. However, a causal relationship between subcellular localization of PLG-Rs in tumor cells and overall patient survival remains to be clarified. Future studies focusing on the processes controlling trafficking of cytosolic PLG-Rs across the cell membrane and their exosome-based release may provide the prerequisite understanding necessary to develop novel therapeutic strategies to treat human cancer.
